# Supplementary material for: Risk Stratification to Guide Prevention and Control Strategies for Arboviruses Transmitted by Aedes aegypti
Source: Trop Med Infect Dis. 2023 Jul 14;8(7):362. doi: 10.3390/tropicalmed8070362 (PMC10386430; doi:10.3390/tropicalmed8070362)
Supplement: Supplementary file 1 [file tropicalmed-08-00362-s001.zip › tropicalmed-2454388-supplementary.pdf]

**Supplementary information**

**Table S1.** Weekly distribution of dengue cases in Tartagal, Salta (Argentina) from 2010 to 2020. (a) For 2016 recorded cases for both dengue and chikungunya are included.

| Week | 2010 | 2011 | 2012 | 2013 | 2014 | 2015 | 2016 | 2017 | 2018 | 2019 | 2020 |
|---|---|---|---|---|---|---|---|---|---|---|---|
| **1** | 0 | 0 | 0 | 0 | 0 | 0 | 4 | 0 | 0 | 0 | 3 |
| **2** | 0 | 0 | 0 | 0 | 0 | 0 | 1 | 0 | 0 | 0 | 2 |
| **3** | 0 | 0 | 0 | 1 | 0 | 0 | 1 | 0 | 0 | 0 | 7 |
| **4** | 0 | 0 | 0 | 0 | 0 | 0 | 2 | 0 | 1 | 3 | 6 |
| **5** | 0 | 0 | 0 | 0 | 0 | 0 | 11 | 0 | 2 | 7 | 6 |
| **6** | 0 | 0 | 0 | 0 | 3 | 0 | 32 | 0 | 2 | 15 | 12 |
| **7** | 0 | 0 | 0 | 1 | 3 | 0 | 14 | 0 | 3 | 16 | 9 |
| **8** | 0 | 0 | 0 | 7 | 1 | 0 | 23 | 0 | 1 | 27 | 0 |
| **9** | 0 | 1 | 0 | 6 | 2 | 0 | 23 | 1 | 2 | 16 | 31 |
| **10** | 0 | 2 | 7 | 19 | 0 | 0 | 16 | 0 | 4 | 9 | 33 |
| **11** | 0 | 9 | 5 | 27 | 2 | 0 | 4 | 1 | 6 | 18 | 32 |
| **12** | 0 | 6 | 2 | 37 | 0 | 0 | 6 | 2 | 9 | 26 | 41 |
| **13** | 0 | 4 | 2 | 18 | 2 | 0 | 11 | 5 | 3 | 14 | 51 |
| **14** | 0 | 5 | 3 | 10 | 4 | 0 | 14 | 7 | 3 | 23 | 87 |
| **15** | 0 | 4 | 2 | 21 | 5 | 0 | 7 | 9 | 1 | 29 | 47 |
| **16** | 0 | 0 | 5 | 11 | 10 | 0 | 14 | 10 | 0 | 35 | 49 |
| **17** | 0 | 0 | 3 | 10 | 12 | 0 | 12 | 8 | 3 | 12 | 41 |
| **18** | 0 | 1 | 1 | 0 | 7 | 0 | 6 | 0 | 0 | 0 | 48 |
| **19** | 0 | 0 | 2 | 2 | 12 | 0 | 8 | 3 | 0 | 0 | 15 |
| **20** | 0 | 1 | 6 | 4 | 8 | 0 | 6 | 1 | 0 | 0 | 0 |
| **21** | 0 | 3 | 0 | 1 | 6 | 0 | 1 | 0 | 0 | 0 | 0 |
| **22** | 0 | 0 | 1 | 1 | 2 | 0 | 0 | 0 | 0 | 0 | 0 |
| **23** | 0 | 0 | 0 | 0 | 1 | 0 | 0 | 0 | 0 | 0 | 0 |
| **24** | 0 | 0 | 0 | 0 | 0 | 0 | 0 | 0 | 0 | 0 | 0 |
| **25** | 0 | 0 | 0 | 0 | 0 | 0 | 0 | 0 | 0 | 0 | 0 |
| **Total** | **0** | **36** | **39** | **176** | **80** | **2** | **216** | **47** | **40** | **250** | **520** |

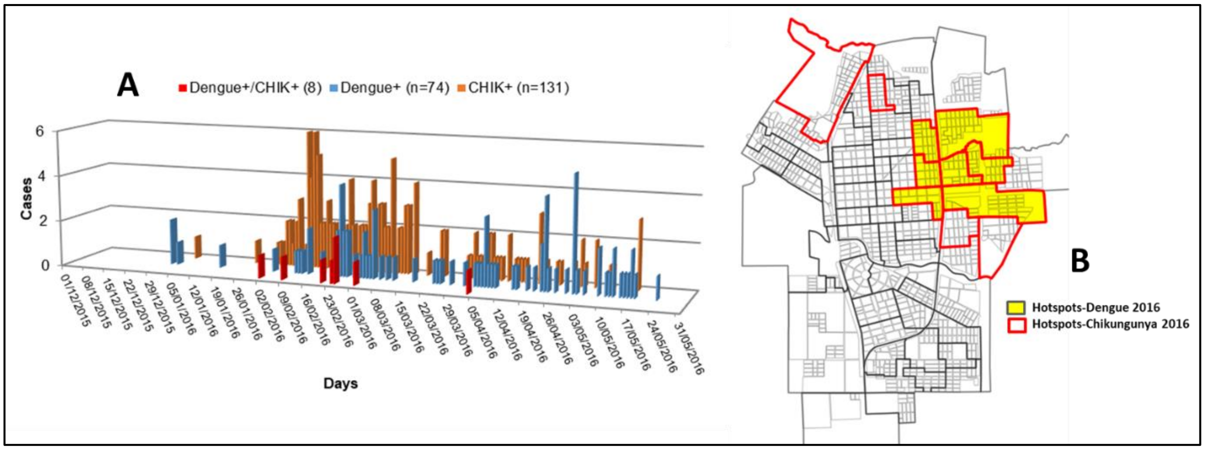


**Supplementary Figure S1.** (A) Weekly evolution of dengue and chikungunya cases in Tartagal, Salta (Argentina) during the 2016 outbreak, (B) Overlap of dengue and chikungunya hotspots.

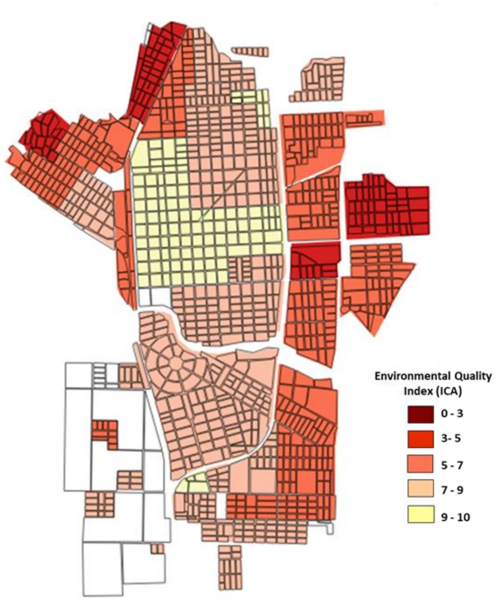


Environmental Quality Index (ICA)

| Neighborhood | ICA < 7 | Neighborhood | ICA > 7 |
|---|---|---|---|
| Sachapera | 3,30 | Tupy Guaraní | 7,10 |
| Chorotes | 3,40 | Santa María 1 | 7,53 |
| La Loma | 4,10 | San Cayetano | 7,61 |
| 9 de Julio | 4,90 | Luján | 7,64 |
| Tomás Sánchez | 5,20 | Fátima | 7,80 |
| San Silvestre | 5,30 | 365 Viviendas | 8,00 |
| Belgrano | 5,79 | El Milagro | 8,00 |
| Santa rita | 5,79 | 104 Viviendas | 8,00 |
| Santa María 2 | 5,90 | Jardines de San Martín | 8,00 |
| Ferroviario | 6,00 | Los Payos | 8,40 |
| Tobas | 6,20 | Villa Zaavedra | 8,55 |
| San Antonio | 6,40 | 203 Viviendas | 8,60 |
| Norte Grande | 6,40 | 128 Viiviendas | 8,60 |
| Roberto Romero y TGN | 6,40 | Villa Güemes | 8,70 |
| Tomás Ryan | 6,70 | Néstor Kirchner | 8,70 |
| Nva. Esperanza | 6,85 | Virgen de la Peña | 8,70 |
| Cherenta | 6,90 | Adolfo Güemes | 8,70 |
| San Juan | 6,90 | San José | 8,80 |
| | | Bicentenario | 8,83 |
| | | Lugones | 8,90 |
| | | SUPE | 8,97 |
| | | San Roque | 9,05 |
| | | 200 Viviendas | 9,10 |
| | | San Isidro | 9,20 |
| | | Alberdi | 9,27 |
| | | Centro | 9,30 |
| | | Santa María 3 | ----- |

**Figure S2.** Environmental quality index per neighborhood in Tartagal, Salta (Argentina) according to the municipality´s strategic plan (24).

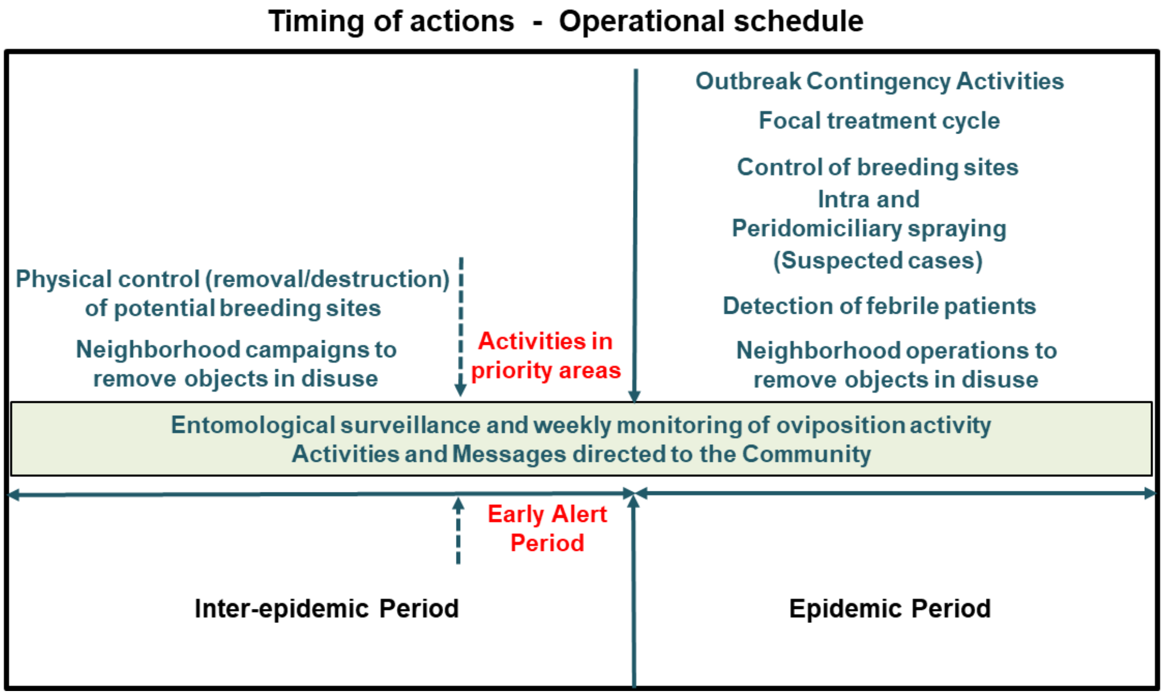


**Figure S3.** Operational scheme for the activities developed throughout the year as part of the Surveillance and Control Program for *Aedes aegypti* implemented in the city of Tartagal, Salta

(Argentina). This chronogram is based on the local epidemiological profile and the pattern of activity and infestation of the vector. The identified hotspots areas have priority status for the implementation of prevention strategies during the inter-epidemic period and mainly during the early alert period of the following season. During the inter-epidemic period activities include the physical control of potential breeding sites through domiciliary visits to identify the sties and remove or destroy them and two-yearly city-wide campaigns to remove unused objects that serve as potential breeding sites. These activities are also performed during the epidemic period, and additional activities centered on focal treatment rounds are included with the use of biolarvicides. When a febrile case is detected, a spot blockade is performed which consists in spraying both the intra and peridomicile of the house and neighboring houses in the same block and the surrounding eight blocks around it. During this spot blockade, the potential breeding sites are controlled with biolarvicide. All these activities are reinforced throughout the entire year with entomological surveillance and weekly monitoring of oviposition activity, together with awareness messages and activities in the community.
